# Changes in Perceptions of Discrimination in Health Care in California, 2003 to 2017

**DOI:** 10.1001/jamanetworkopen.2019.6665

**Published:** 2019-07-03

**Authors:** Lucy B. Schulson, Michael K. Paasche-Orlow, Ziming Xuan, Alicia Fernandez

**Affiliations:** 1 Section of General Internal Medicine, Boston University School of Medicine, Boston, Massachusetts; 2 Boston Medical Center, Boston, Massachusetts; 3 Department of Community Health Sciences, Boston University School of Public Health, Boston, Massachusetts; 4 Division of General Internal Medicine, University of California, San Francisco; 5 Zuckerberg San Francisco General Hospital and Trauma Center, San Francisco, California

## Abstract

**Importance:**

Research in the early 2000s in California demonstrated that racial and ethnic minorities, immigrants, and those with limited English proficiency (LEP) experienced high rates of discrimination in health care. Less is known about how patients’ perceptions of discrimination in health care have changed since then.

**Objective:**

To determine whether perceptions of discrimination in health care have changed overall and for specific vulnerable populations.

**Design, Setting, and Participants:**

This cross-sectional study used data from the California Health Interview Survey for state residents aged 18 years and older for 2 periods, 2003 to 2005 and 2015 to 2017. χ^2^ analyses and multivariate logistic regression were performed to compare recent discrimination in health care in late vs early periods controlling for race/ethnicity, poverty level, education, insurance status, usual source of care, self-reported health, and LEP. Additional subanalyses were performed by race/ethnicity, immigrant status, and LEP status. Jackknife replicate weights were provided by the California Health Interview Survey.

**Exposure:**

Survey year was dichotomized as combined 2003 to 2005 and combined 2015 to 2017.

**Main Outcomes and Measures:**

Survey respondents were identified as having experienced recent discrimination in health care if they responded “yes” to the question, “Was there ever a time when you would have gotten better medical care if you had belonged to a different race or ethnic group?” and reported that this occurred within the last 5 years.

**Results:**

There were 84 088 participants in 2003 to 2005 (51.0% female; 14.7% aged ≥65 years) and 63 242 participants in 2015 to 2017 (51.1% female; 18.0% aged ≥65 years). Rates of recent discrimination in health care decreased from 6.0% to 4.0% (difference, 2.0%; 95% CI, 1.5%-2.5%; *P* < .001). In adjusted analyses, perceptions of discrimination in health care decreased in 2015 to 2017 compared with 2003 to 2005 (odds ratio [OR], 0.60; 95% CI, 0.53-0.68; *P* < .001). There was a significant race × period interaction for Latino individuals (OR, 0.58; 95% CI, 0.40-0.83; *P* = .003) but not for Asian individuals (OR, 0.76; 95% CI, 0.50-1.16; *P* = .20) or African American individuals (OR, 1.24; 95% CI, 0.76-2.02; *P* = .40). There was a significant immigrant status × period interaction (OR, 0.55; 95% CI, 0.44-0.69; *P* < .001) and LEP status × period interaction (OR, 0.67; 95% CI, 0.51-0.89; *P* < .001).

**Conclusions and Relevance:**

This study suggests that perceptions of discrimination in health care in California decreased between 2003 to 2005 and 2015 to 2017 among Latino individuals, immigrants, and those with LEP. African American participants reported consistently high rates of discrimination, indicating that interventions targeting health care discrimination are still necessary.

## Key Points

**Question:**

Have perceptions of discrimination in health care changed in California over the last decade?

**Findings:**

This repeated cross-sectional study of a racially, ethnically, and linguistically diverse adult population using results from the California Health Interview Survey found a significant overall decrease in perceptions of discrimination in health care (from 6.0% to 4.0%). In subanalyses this finding was significant among Latino respondents, immigrants, and those with limited English proficiency; however, perceptions of discrimination in health care among African American individuals have not improved and remain relatively high.

**Meaning:**

This study suggests that perceptions of discrimination in health care have improved for some populations, but interventions to reduce discrimination in health care are still necessary.

## Teaser

This cross-sectional study investigates changes in perceptions of discrimination in health care on the basis of race/ethnicity, immigration status, and English proficiency in California from 2003 to 2017.

## Introduction

Perception of discrimination in health care is associated with lower quality of life, worse mental health outcomes, and poorer physical health and mediates racial and ethnic health disparities.^1^ The California Health Interview Survey (CHIS) included questions about discrimination in health care in 2003 and 2005, and analyses of CHIS data from those years found that participants who reported nonwhite race/ethnicity, were immigrants, or had limited English proficiency (LEP) were more likely to report discrimination in health care.^2,3,4,5^ This question on discrimination in health care was removed from the survey after 2005 but then reintroduced in 2015.

Over the last 10 years, California’s health care community and legislature have made concerted efforts to address health care disparities. We hypothesized that perceptions of discrimination in health care will have decreased over time overall and for all subgroups of the population. To determine whether perceptions of discrimination in health care have changed over time in California, we performed a repeated cross-sectional study comparing data from the 2003 to 2005 and 2015 to 2017 CHIS.

## Methods

### Data Source

The CHIS, conducted biennially since 2001 and annually since 2011, includes approximately 20 000 adult respondents sampled from the residents of California each year.^6,7,8,9^ The telephone-based survey uses a dual-frame random digit dial technique, and 1 adult in each randomly sampled participating household is interviewed.^10,11,12,13^ In 2003 to 2005, surveys were conducted in English, Spanish, Mandarin, Cantonese, Vietnamese, and Korean.^10,11^ For 2015 to 2017, Tagalog was added.^12,13^ In the CHIS design, missing values are replaced through imputation using either random selection or hot deck imputation. In 2017, external adjustment was also used for missing values.^14,15,16,17^ Participation in the survey is voluntary and all participants give verbal informed consent.^18^ The response rate of the adult sample was 59.9% in 2003, 54% in 2005, 47.9% in 2015 to 2016, and 66.6% in 2017.^19,20,21,22^ Since 2007, CHIS has included residents of cell phone–only households. In survey years 2015 to 2017, approximately 50% of the interviews were conducted from cell phones.^12,13^ This study was deemed exempt by the institutional review board at the Boston University School of Medicine. This study followed the Strengthening the Reporting of Observational Studies in Epidemiology (STROBE) reporting guideline.

### Recent Discrimination in Health Care

We identified experience of recent discrimination in health care if a participant answered “yes” to the question, “Was there ever a time when you would have gotten better medical care if you had belonged to a different race or ethnic group?” and also reported that the discrimination occurred within the last 5 years. This question was included in the 2003, 2005, 2015, 2016, and 2017 survey waves but not during the intervening years. This item was previously used in research on discrimination in health care in California in the early 2000s and is very similar to the Commonwealth Fund 2001 Health Care Quality Survey question on discrimination in health care.^3,5,23,24^

### Exposures of Interest

Our primary exposure of interest was survey period, dichotomized as combined 2003 to 2005 and combined 2015 to 2017. Respondents were categorized based on self-reported race/ethnicity as Latino, Asian, non-Latino African American, and non-Latino white. Those who identified as American Indian or Alaska Native, more than 2 races, or other were categorized as other. Respondents who were born outside the United States were coded as immigrant. Respondents who stated they spoke English not well or not at all were included in the LEP group. Those who spoke only English or spoke English well or very well were considered English proficient.

### Covariates

Age was defined by an ordinal variable (18-29, 30-39, 40-49, 50-64, or ≥65 years). We accounted for socioeconomic factors by including income relative to the poverty level (0%-99%, 100%-199%, 200%-299%, and ≥300% of poverty level), education status (less than high school or no formal education; high school graduate, generalized education diploma, or vocation school; some college or associate’s degree; and college graduate and beyond), and insurance status (uninsured, any Medicaid or public insurance, Medicare, and commercial insurance). Usual source of care was also included in the models (doctor’s office, community or government clinic, emergency department or urgent care, some other place or no particular place, and no usual source of care), as was self-reported health (excellent, very good, good, fair, and poor).

### Statistical Analysis

We used jackknife replicate weights provided by the CHIS to reflect the population of California.^9^ We performed χ^2^ analyses comparing perceptions of recent discrimination in 2003 to 2005 vs 2015 to 2017 overall, by race/ethnicity, immigration status, and LEP status. We then performed multivariate logistic regression controlling for self-reported race/ethnicity, sex, poverty level, education, insurance status, usual source of care, self-reported health, and LEP. We also performed subanalyses using a period interaction term by racial/ethnic groups, immigrant status, and LEP status. Because a national study demonstrated a decrease in perceived discrimination in health care for African American individuals with chronic disease, we performed an additional set of analyses limited to those with reported fair to poor health (eTable 1 in the Supplement).^25^ We also performed a logistic regression stratified by period to determine whether covariates associated with discrimination in 2003 to 2005 changed in 2015 to 2017 (eTable 2 in the Supplement). In addition, we ran exploratory analyses comparing 2015 to 2017 (eTable 3 in the Supplement). All analyses were 2-tailed and used a significance level of *P* < .05. Analyses were performed using SAS statistical software version 9.4 (SAS Institute Inc).

## Results

There were 84 088 participants (51.0% female) in 2003 to 2005 and 63 242 participants (51.1% female) in 2015 to 2017. Data for the primary dependent variable were missing for less than 5% (616 participants). Table 1 presents data of sample characteristics weighted to the population of California. Comparing 2003 to 2005 with 2015 to 2017, there were more participants who identified as Latino and Asian in the later period (30.7% vs 35.5%, 11.6% vs 14.3%, respectively). The later period included more participants aged 65 years or older (14.7% vs 18.0%). Fewer people had a usual source of care in the later period (13.5% vs 15.2%) and fewer people were uninsured (16.6% vs 9.4%). There was no statistically significant difference in proportion of immigrants (33.5% vs 33.3%). Compared with the early years, fewer individuals had LEP in the sample in later years (16.2% vs 15.0%). Perceptions of recent discrimination decreased overall (6.0% in 2003-2005 vs 4.0% in 2015-2017; difference, 2.0%; 95% CI, 1.5%-2.5%; *P* < .001). Rates decreased between the early period and later period among Latino participants (11.0% vs 5.4%; difference, 5.6%; 95% CI, 4.6%-6.5%; *P* < .001) and Asian participants (6.4% vs 4.0%; difference, 2.5%; 95% CI, 0.95%-4.0%; *P* = .01). In addition, compared with the early period, discrimination in health care was reported by a smaller proportion of immigrants (10.6% vs 5.2%; difference, 5.3%; 95% CI, 4.4%-6.3%; *P* < .001) and LEP individuals (13.6% vs 6.5%; difference, 7.1%; 95% CI, 5.5%-8.8%; *P* < .001) in the later period. By contrast, rates were unchanged between the 2 periods among white individuals (2.1% vs 1.8%; difference, 0.3%; 95% CI, −0.1% to 0.9%; *P* = .25) and African American individuals (10.7% vs 9.9%; difference, 0.7%; 95% CI, −2.6% to 4.1%; *P* = .67) (Figure).

**Table 1.** Population Characteristics in 2003 to 2005 and 2015 to 2017

Variable	%		*P* Value
	2003-2005 (N = 84 088)^a^	2015-2017 (N = 63 242)^a^	
Race/ethnicity			
White	48.8	41.6	<.001^b^
African American	6.1	5.6	
Asian	11.6	14.3	
Latino	30.7	35.5	
Other^c^	2.8	3.1	
Sex			
Male	49.0	48.9	<.001^b^
Female	51.0	51.1	
Age, y			
18-29	23.0	22.1	<.001^b^
30-39	21.2	18.0	
40-49	20.7	17.2	
50-64	20.4	24.7	
≥65	14.7	18.0	
Income as % of federal poverty level			
0%-99%	15.0	17.0	.001^b^
100%-199%	18.9	18.5	
200%-299%	14.0	13.4	
≥300%	52.1	51.1	
Education level			
Less than high school or no education	20.3	16.8	<.001^b^
High school graduate or vocational school	23.8	24.2	
Some college	17.6	20.9	
College graduate	38.3	38.1	
General health condition			
Excellent	21.5	17.9	<.001^b^
Very good	29.7	30.2	
Good	28.3	30.8	
Fair	15.8	16.7	
Poor	4.8	4.5	
Usual source of care			
Physician’s office	68.7	58.1	<.001^b^
Community or government clinic	15.2	24.0	
Emergency department or urgent care	1.8	1.7	
Some other place or no particular place	0.7	1.0	
No usual source of care	13.5	15.2	
Insurance type			
Uninsured	16.6	9.4	<.001^b^
Medicaid or public insurance	15.1	28.2	
Medicare	11.6	13.5	
Commercial	56.7	48.9	
Immigrant			
Yes	33.5	33.3	.70
No	66.5	66.7	
Limited English proficiency			
Yes	16.2	15.0	.02^b^
No	83.8	85.0	

^a^ Numbers are of survey sample, but percentages are weighted to reflect California’s population.

^b^ Significant at a *P* value of .05.

^c^ Other race included American Indian or Alaska Native, more than 2 races, or another race.

**Figure.**
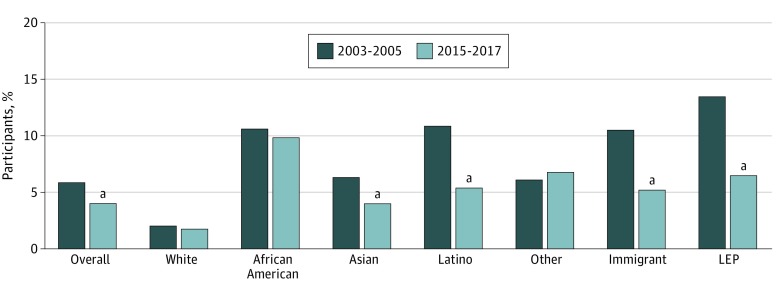
Unadjusted Rates of Perceptions of Discrimination in Health Care in 2003 to 2005 vs 2015 to 2017 by Race/Ethnicity, Immigration Status, and Limited English Proficiency (LEP) ^a^Indicates a statistically significant result at *P* < .05.

In adjusted analyses, perceptions of discrimination in health care decreased in 2015 to 2017 compared with 2003 to 2005 (adjusted odds ratio [aOR], 0.60; 95% CI, 0.53-0.68; *P* < .001). This association was significant for Latino respondents (aOR, 0.58; 95% CI, 0.40-0.83; *P* = .003) but did not reach statistical significance for Asian respondents (aOR, 0.76; 95% CI, 0.50-1.16; *P* = .20) or African American participants (aOR, 1.24; 95% CI, 0.76-2.02; *P* = .40). There was a significant immigration status × period interaction (OR, 0.55; 95% CI, 0.44-0.69; *P* < .001) and LEP status × period interaction (OR, 0.67; 95% CI, 0.51-0.89; *P* < .001) (Table 2). Sensitivity analyses demonstrated consistent results for participants with poorer health (eTable 1 in the Supplement). Results were also consistent when examined separately by period (eTable 2 in the Supplement) and there were no significant differences between 2015 and 2017 (eTable 3 in the Supplement).

**Table 2.** Perceived Recent Discrimination in Health Care in 2003 to 2005 vs 2015 to 2017 Controlling for Demographic Covariates

Model or Covariate	Adjusted OR (95% CI)^a^	*P* Value
Model 1^b^		
Overall	0.60 (0.53-0.68)	<.001^c^
Model 2: race × period interaction term^d^		
African American	1.24 (0.76-2.02)	.40
Asian	0.76 (0.50-1.16)	.20
Latino	0.58 (0.40-0.83)	.003^c^
Other	1.27 (0.84-1.93)	.27
Model 3: immigrant × period interaction term^e^		
Immigrant	0.55 (0.44-0.69)	<.001^c^
Model 4: LEP × period interaction term^f^		
LEP	0.67 (0.51-0.89)	<.001^c^

Abbreviations: LEP, limited English proficiency; OR, odds ratio.

^a^ Probability of recent discrimination in health care in later period (2015-2017) compared with earlier period (2003-2005), with earlier period as reference.

^b^ Model 1 includes race, sex, age, education, poverty level, insurance status, general health, usual source of care, and LEP.

^c^ Significant at *P* < .05.

^d^ Model 2 includes sex, age, education, poverty level, insurance status, general health, usual source of care, and LEP. Race is included in the model as an interaction term. White race is the reference.

^e^ Model 3 includes race, sex, age, education, poverty level, insurance status, general health, usual source of care, and time in the United States. Immigrant status is an interaction term, with nonimmigrant status as the reference.

^f^ Model 4 includes race, sex, age, education, poverty level, insurance status, general health, usual source of care, and time in the United States. Limited English proficiency is an interaction term, with English speaking as the reference.

## Discussion

Using a statewide representative data set, we found that reports of recent discrimination in health care in California decreased substantially in 2015 to 2017 compared with 2003 to 2005 for Latino individuals, immigrants, and people with LEP but not for African American individuals.

We can only speculate as to why discrimination decreased for some populations and not for others in the decade between 2005 and 2015 in California, but several possible factors are worth noting. First, the enormous growth of the state’s Latino population, currently at 39%, led to increased political representation at multiple levels of government as noted by the election in 2005 of the first Latino mayor of Los Angeles.^26,27^ Greater civic and social inclusion may affect perceptions of discrimination in general, including in health care. Second, passage of the Patient Safety and Affordable Care Act in 2010 resulted in a decrease of the uninsured population in California from 19% in 2010 to 7% in 2017, with the greatest insurance gains occurring among Latino individuals.^28^ Although a recent study by Alcalá and Cook^23^ found that having Medicaid insurance as opposed to employer-sponsored insurance was associated with perceived discrimination, access to insurance may reduce perceptions of discrimination in health care among the previously uninsured. That said, as we included insurance status in our adjusted model, insurance status alone is unlikely to explain our findings.

Changes in statewide policies may also partly explain our findings. The Health Care Language Assistance Act,^29^ which holds health plans accountable for the provision of language services and requires health plans and health insurers to provide their enrollees with interpreter services and translated material and to collect data on race, ethnicity, and language, was passed in 2003 and went into full effect in 2009.^30^ The Health Care Interpreter Network,^31^ which provides video and voice interpretation to public hospitals and clinics that serve the California Medicaid population, was developed in 2005.^32^ Provision of language services can signal even to English-speaking patients that all are welcome. In 2012, the state Office of Health Equity was established and made workforce diversity in California a priority.^33^ Increased diversity among students and trainees may have influenced the culture of health care. While the policies themselves may not have changed the health care climate, they are markers of increased consciousness regarding health equity and justice.

Our findings differ from those of Nguyen et al,^25^ who found national declines in patient-reported discrimination among African American individuals and no change among Latino individuals between 2008 and 2014. Participants in the study by Nguyen and colleagues were patients with chronic disease. Our analysis of participants reporting poorer health was consistent with our overall findings, suggesting that the African American and Latino experiences are different in California than they are nationally.

### Limitations

There are several limitations to our work. First, our study was limited to California. Demographic characteristics, health care access, and statewide health care initiatives differ substantially in other states. Second, we do not know whether our findings represent a linear trend because surveys between 2005 and 2015 did not include questions on discrimination in health care. In fact, the current political climate, particularly around immigration, may lead to a resurgence of perceived discrimination among immigrants and people with LEP.^34^

## Conclusions

Perceptions of discrimination in health care in California decreased significantly overall between 2003 and 2017, a change that was most notable among Latino individuals, immigrants, and LEP individuals. Some of these changes are likely due to explicit statewide efforts to address discrimination, which may serve as a blueprint for other states. Research is needed to understand why these efforts fell short in addressing discrimination against African American individuals. It is critical to continue to examine and address perceptions of discrimination in health care.

## Notes

Supplement. **eTable 1.** Perceived Recent Discrimination in Healthcare Among Those With Fair to Poor Health in 2015-2017 vs. 2003-2005 Controlling for Demographic Covariates **eTable 2.** Adjusted Results of Predictors of Recent Discrimination in Healthcare in 2003-2005 and 2015-2017 **eTable 3.** Perceived Recent Discrimination in Healthcare in 2015 vs. 2017 Controlling for Demographic Covariates
