# Supplementary material for: Changes in Perceptions of Discrimination in Health Care in California, 2003 to 2017
Source: JAMA Netw Open. 2019 Jul 3;2(7):e196665. doi: 10.1001/jamanetworkopen.2019.6665 (PMC6613287; doi:10.1001/jamanetworkopen.2019.6665)
Supplement: Supplement. — eTable 1. Perceived Recent Discrimination in Healthcare Among Those With Fair to Poor Health in 2015-2017 vs. 2003-2005 Controlling for Demographic Covariates eTable 2. Adjusted Results of Predictors of Recent Discrimination in Healthcare in 2003-2005 and 2015-2017 eTable 3. Perceived Recent Discrimination in Healthcare in 2015 vs. 2017 Controlling for Demographic Covariates [file jamanetwopen-2-e196665-s001.pdf]

Supplementary Online Content

Schulson LB, Paasche-Orlow MK, Xuan Z, Fernandez A. Changes in perceptions of discrimination in health care in California, 2003 to 2017. *JAMA Netw Open*. 2019;2(7):e196665. doi:10.1001/jamanetworkopen.2019.6665

eTable 1. Perceived Recent Discrimination in Healthcare Among Those With Fair to Poor Health in 2015-2017 vs. 2003-2005 Controlling for Demographic Covariates

eTable 2. Adjusted Results of Predictors of Recent Discrimination in Healthcare in 2003-2005 and 2015-2017

eTable 3. Perceived Recent Discrimination in Healthcare in 2015 vs. 2017 Controlling for Demographic Covariates

This supplementary material has been provided by the authors to give readers additional information about their work.

© 2019 Schulson LB et al. *JAMA Network Open.*

**eTable 1:** Perceived Recent Discrimination in Healthcare among those with Fair to Poor Health in 2015-2017 vs. 2003-2005 controlling for Demographic Covariates			
	**Adjusted OR[a]**	**95% CI**	**p-value**
**Model 1[b]**			
Overall	0.60	0.50, 0.73	<.0001
**Model 2: Race by Time Period Interaction Term [c]**			
African-American	0.94	0.34, 2.64	0.91
Asian	0.87	0.41, 1.86	0.72
Latino	0.37	0.0.23,0.67	.0007
Other	1.46	0.63, 3.35	0.37

[a] Probability of recent discrimination in health care in late years compared to early years, with early years as reference.
[b] Model 1 includes race, sex, age, education, poverty level, insurance status, general health, usual source of care, and LEP
[c] Model 2 includes sex, age, education, poverty level, insurance status, general health, usual source of care, and LEP. Race is included in the model as an interaction term. White is referent.

**eTable 2: Adjusted Results of Predictors of Recent Discrimination in Healthcare in 2003-2005 and 2015-2017**						
	**2003-2005**			**2015-2017**		
	**Adjusted OR[a]**	**95% CI**	**p-value**	**Adjusted OR[a]**	**95% CI**	**p-value**
Latino [b]	2.66	2.34, 3.03	<.0001	1.96	1.27, 3.03	.0025
Asian [b]	2.41	2.04,2.85	<.0001	1.89	1.24, 2.88	.0030
African-American [b]	4.01	3.43, 4.68	<.0001	4.50	2.75, 7.40	<.0001
Other	2.15	1.77,2.62	<.0001	3.02	1.97, 4.62	<.0001
LEP [c]	1.11	0.96, 1.29	0.1741	1.14	0.76, 1.75	0.55
Immigrant	1.53	1.37, 1.71	<.0001	1.20	0.84, 1.70	0.32
Male Sex	1.03	0.95, 1.11	0.5321	1.29	0.97, 1.72	0.08
0-99% poverty [d]	2.10	1.80, 2.45	<.0001	1.83	1.21, 2.76	.0044
Less than high school education [e]	0.89	0.71, 1.14	0.1651	0.69	0.38, 1.28	0.24
Poor Health [f]	2.62	2.13, 3.22	<.0001	2.82	1.61, 4.90	.0002
Uninsured [g]	1.35	1.20, 1.53	<.0001	1.25	0.88, 1.77	0.22
Medicaid [g]	1.12	0.98, 1.28	0.1022	1.71	1.24, 2.40	.0011
Community Health Center [h]	1.28	1.13, 1.46	0.0002	1.09	0.83, 1.44	0.52

© 2019 Schulson LB et al. *JAMA Network Open.*

Emergency department/ Urgent care [h]	1.27	0.98, 1. 64	0.07	1.60	0.98, 2.64	0.06
Some other place/ no particular place [h]	2.94	2.09, 4.14	<.0001	1.51	0.66, 3.46	0.33
No usual source of care [h]	1.29	1.11, 1.50	.0008	1.05	0.75, 1.47	0.78

[a] Probability of recent discrimination in health care controlling for race/ethnicity, LEP status, sex, poverty level, education, health status, insurance type, usual source of race, and age. In models where immigration status is included, LEP status was excluded due to collinearity.

[b] White as referent

[c] Limited English Proficiency

[d] > 300% poverty level as referent

[e] College graduate as referent

[f] Excellent health as referent

[g] Commercial insurance as referent

[h] Doctor's office as referent

eTable 3: Perceived Recent Discrimination in Healthcare in 2015 vs. 2017 Controlling for Demographic Covariates

	**Adjusted OR[a]**	**95% CI**	**p-value**
**Model 1[b]**			
Overall	1.06	0.82, 1.36	0.67
**Model 2: Race by Time Period Interaction Term [c]**			
African-American	1.18	0.31. 4.46	0.81
Asian	1.43	0.59, 3.48	0.26
Latino	1.15	0.45, 2.92	0.77
Other	1.65	0.68, 3.97	0.26
**Model 3: Immigrant by Time Period Interaction Term [d]**			
Immigrant by Year Interaction Term	0.97	0.59, 1.59	0.91
**Model 4: LEP [e] by Time Period Interaction Term [f]**			
LEP [e] by Year Interaction Term	1.01	0.60, 1.71	0.96

[a] Probability of recent discrimination in health care in 2015 compared to 2017, with 2015 as reference.

[b] Model 1 includes race, sex, age, education, poverty level, insurance status, general health, usual source of care, and LEP

[c] Model 2 includes sex, age, education, poverty level, insurance status, general health, usual source of care, and LEP. Race is included in the model as an interaction term. White is referent.

[d] Model 3 includes race, sex, age, education, poverty level, insurance status, general health, usual source of care, and time in the USA. Immigrant is an interaction term with non-immigrant as referent.
[e] Limited English Proficiency
[f] Model 4 includes race, sex, age, education, poverty level, insurance status, general health, usual source of care, and time in the USA. LEP is an interaction term with English speaking as referent.

© 2019 Schulson LB et al. *JAMA Network Open.*
